# Time Course and Microcircuit Mechanisms of Primary Motor Cortex Dysfunction in Progressive Parkinsonism

**DOI:** 10.64898/2026.07.27.741031

**Published:** 2026-07-30

**Authors:** Liqiang Chen, Hiba Douja Chehade, Samhitha Somavarapu, Hong-Yuan Chu

## Abstract

Dysfunction of the primary motor cortex (M1) has long been implicated in the pathophysiology of Parkinson’s disease (PD), mostly as a link by which abnormal basal ganglia and thalamic activities are translated into motor symptoms. However, emerging evidence suggests that M1 neurons also exhibit maladaptive changes in advanced parkinsonism. Here, using a progressive mouse model of nigrostriatal neurodegeneration (i.e., the MitoPark mice, MP), we found that M1 neurons develop age- and striatal dopamine-dependent synaptic and cellular adaptations as parkinsonism progresses. The optogenetic, electrophysiological, pharmacological, and CRISPR-mediated genetic studies demonstrated that impaired α5-GABA _A_ receptor-mediated inhibition and excessive activation of NMDA receptors of M1 pyramidal neurons are key microcircu it mechanisms underlying cortical circuit remodeling during progressive striatal dopamine (DA) loss. Furthermore, we found that treatment with L-DOPA at early or late stages of parkinsonism can prevent or rescue, respectively, the synaptic and cellular adaptations in M1. Together, the present study demonstrates the time course and the underlying molecular and microcircuit mechanisms of cortical network dysfunction during the development of parkinsonism.

## Full Text Availability

The license terms selected by the author(s) for this preprint version do not permit archiving in PMC. The full text is available from the preprint server.

